# Interactive Association of Five Candidate Polymorphisms in Apelin/APJ Pathway with Coronary Artery Disease among Chinese Hypertensive Patients

**DOI:** 10.1371/journal.pone.0051123

**Published:** 2012-12-03

**Authors:** Wei Jin, Xiuxiu Su, Min Xu, Yan Liu, Jingyi Shi, Lin Lu, Wenquan Niu

**Affiliations:** 1 Department of Cardiology, Ruijin Hospital, Shanghai Jiao Tong University School of Medicine, Shanghai, China; 2 State Key Laboratory of Medical Genomics, Ruijin Hospital, Shanghai Jiao Tong University School of Medicine, Shanghai, China; 3 Shanghai Institute of Hypertension, Shanghai, China; Tor Vergata University of Rome, Italy

## Abstract

**Background:**

Via sequencing the genes of apelin/angiotensin receptor-like 1 (apelin/APJ) pathway, we have recently identified and validated four common polymorphisms (rs3761581, rs56204867, rs7119375, and rs10501367) implicated in the development of hypertension. Extending these findings, we, in Chinese hypertensive patients, sought to investigate the association of these four polymorphisms and one additional promising candidate (rs9943582) from this pathway with the risk of developing coronary artery disease (CAD).

**Methodology/Principal Findings:**

Genotypes were obtained from 994 sporadic CAD patients and 708 age- and sex-matched controls. All participants were hypertensives and angiographically-confirmed. Data were analyzed by Haplo.Stats and multifactor dimensionality reduction (MDR) softwares. Genotype distributions of five examined polymorphisms satisfied Hardy-Weinberg equilibrium in controls of both genders. Single-locus analyses exhibited no significant differences in the genotype/allele frequencies of examined polymorphisms between CAD patients and controls (P>0.05), even after controlling traditional cardiovascular confounders. In haplotype analyses, low-penetrance haplotype G-A (in order of rs56204867 and rs3761581 from apelin gene) was significantly overrepresented in controls (1.73%) relative to in CAD patients (0.4%) in males (P = 0.047). Further interaction analyses suggested an overall best MDR model including rs3761581 in males (P = 0.0408) and including rs7119375 and rs9943582 in females (P<0.0001), which were further substantiated in the classical logistical regression model.

**Conclusions:**

Our findings demonstrated a contributive role of low-penetrance haplotype in apelin gene on CAD in males, and more importantly, interactive effects of genetic defects in apelin/APJ pathway might confer a potential risk in Chinese hypertensive patients.

## Introduction

Although investigations from single locus to genome wide association studies are proliferating in the literature, the challenge to unravel the ultimate genetic underpinnings of cardiovascular diseases obsesses global geneticists [1]. The most compelling reason might be attributed to failure to account for multiple genetic interactions. In this context, considering the totality of available evidence, the feasible pathway-based approach that examines interactions of relevant genes may be more informative to promote our understanding of the genetic etiology of complex diseases [2].

The apelin/angiotensin receptor-like 1 (apelin/APJ) pathway has emerged as an essential novel mediator of cardiovascular disease [3], [4]. The APJ is a 7-transmembrane domain receptor that was first cloned in 1993 [5], and its endogenous ligand was isolated in 1998 termed apelin [6]. Since the discovery of the apelin/APJ pathway, numerous functional studies have been conducted to assess the involvement of apelin and APJ in cardiovascular system [7], [8]. Although the discovery of susceptibility loci by high-throughput genomic techniques has shed some light on the fundamental mechanisms that influence disease predisposition [9], genetic data on apelin/APJ pathway and the risk of CAD are sparse in the literature. Recently, we have identified 12 common polymorphisms in apelin/APJ pathway by direct sequencing, and investigated for the first time the association of these polymorphisms with hypertension and its related phenotypes in a family-based association study [10], and we further validated the top four polymorphisms (rs3761581, rs56204867 (T-1860C), rs7119375, and rs10501367) in a large population-based case-control study [11]. Our findings provided strong evidence for the genetic involvement of apelin/APJ pathway in susceptibility to hypertension. As widely accepted, hypertension is an established risk factor for the development of coronary artery disease (CAD), almost doubling its risk [12], [13]. It is therefore reasonable to hypothesize that genetic defects in apelin/APJ pathway, leading to elevated blood pressure, play a pivotal role in the pathogenesis of CAD.

**Figure 1:**
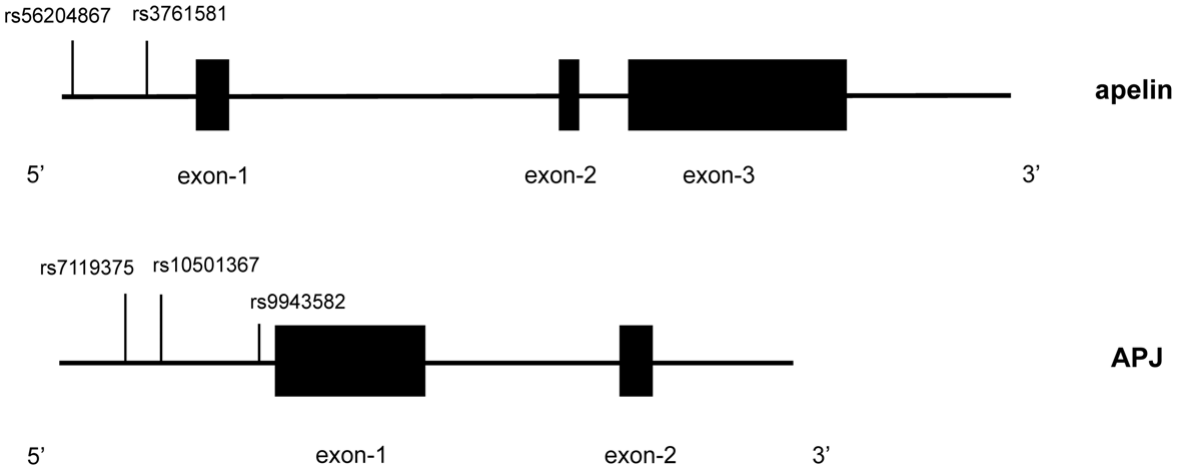
Genomic organization of human apelin (upper panel) and APJ (lower panel) genes, and localization of examined polymorphisms. Each exon is represented by a black box. Lengths of exons and introns are on the proportional scale. The vertical line marks the position of each polymorphism.

To test this hypothesis, we sought to investigate the association of the aforementioned top four polymorphisms and one additional candidate (rs9943582) in APJ gene that was reportedly susceptible to brain infarction [14], with CAD among angiographically-confirmed Chinese hypertensive patients.

**Table 1:** The anthropometric index and clinical biomarkers of the study population between CAD patients and controls according to gender.

Characteristics	Males			Females		
	Patients (n = 533)	Controls (n = 359)	P	Patients (n = 461)	Controls (n = 349)	P
Age^*^, years	60.72 (9.01)	62.66 (9.61)	0.03	65.96 (8.04)	65.1 (10.42)	0.535
BMI, kg/m^2^	25.67 (3.11)	25.25 (3.21)	0.303	24.95 (3.2)	24.92 (3.79)	0.913
SBP, mmHg	135.18 (20.15)	136.95 (18.5)	0.173	139.5 (20.67)	137.36 (17.65)	0.259
DBP, mmHg	81.71 (12.42)	82.5 (11.46)	0.321	80.35 (11.16)	82.63 (10.55)	0.007
Glucose, mmol/L	6.15 (2.13)	5.24 (1.11)	<0.005	6.14 (2.18)	5.41 (1.12)	<0.005
TG, mmol/L	1.83 (0.95)	1.78 (1.04)	0.239	1.98 (1.13)	1.76 (0.89)	0.01
TC, mmol/L	4.37 (1.11)	4.37 (0.84)	0.578	4.83 (1.21)	4.83 (0.95)	0.486
HDL-C, mmol/L	1.02 (0.22)	1.16 (0.36)	<0.005	1.23 (0.28)	1.34 (0.32)	<0.005
LDL-C, mmol/L	2.66 (0.92)	2.57 (0.71)	0.48	2.85 (0.97)	2.85 (0.79)	0.463
Apo A, mmol/L	1.14 (0.18)	1.27 (0.24)	<0.005	1.3 (0.27)	1.38 (0.27)	<0.005
Apo B, mmol/L	0.91 (0.25)	0.86 (0.21)	0.004	0.95 (0.28)	0.94 (0.4)	0.371
Lp(a), mmol/L	0.3 (0.49)	0.2 (0.18)	<0.005	0.27 (0.25)	0.21 (0.19)	0.002
BUN, mmol/L	6.08 (4.28)	6.05 (4.83)	0.969	5.71 (3.38)	5.6 (4.08)	0.276
Cr, μmol/L	97.62 (43.68)	90.38 (27.89)	0.012	75.46 (21.69)	72.52 (18.56)	0.146
UA, μmol/L	346.99 (104.86)	359.2 (91.59)	0.059	308.28 (90.17)	304.34 (90.12)	0.353
hsCRP, mmol/L	17.96 (57.26)	2.22 (4.27)	<0.005	7.24 (14.11)	2.47 (3.4)	<0.005

*Abbreviations*: BMI, body mass index; SBP, systolic blood pressure, DBP, diastolic blood pressure; TC, total cholesterol; TG, triglyceride; HDL-C, high-density lipoprotein cholesterol; LDL-C, low-density lipoprotein cholesterol; Apo A, Apolipoprotein A; Apo B, Apolipoprotein B; Lp(a), Lipoprotein(a); BUN, blood urea nitrogen; Cr, Creatinine; UA, uric acid; hsCRP, high sensitivity C-reactive protein. Continuous variables with skewed distributions were transformed in square for age; in log(10) for SBP, DBP, triglycerides, hsCRP; in square root for BMI, LDL-C, and comparison between CAD patients and controls was conducted using the unpaired t-test. The nonparametric Mann-Whitney U test was used for TC, HDL-C, Apo A, Apo B, Lp(a), glucose, BUN, Cr, and UA. Data are expressed as mean (standard deviation or SD). * For patients, age referred to the onset age of CAD, and for controls, age was recorded at enrollment.

## Materials and Methods

### Study population

This was a hospital-based case-control study encompassing a total of 1702 unrelated Han Chinese who were hypertensive patients and admitted to Ruijin Hospital. All participants were classified into CAD group and control group according to their angiographic results. Coronary angiography was performed using standard Judkins techniques or though radial approach. All major coronary arteries were carefully imaged on at least two orthogonal views. The CAD group enrolled was angiographically-confirmed in the presence of more than 70% stenosis in at least one of the three major coronary arteries or major branches. Patients with simple spasm of coronary arteries, myocardial bridge or other non-coronary atherosclerotic lesions were excluded. The CAD group contained 994 sporadic patients aged 63.15 (standard deviation: 8.96) years with 53.6% male gender. The remaining participants (n = 708), who had normal coronary arteries on angiography, formed age-matched (63.93 (10.09) years) and sex-matched (50.7% male gender) control group.

**Table 2:** Genotype/allele frequencies of five polymorphisms in apelin/APJ system between CAD patients and controls.

Gene: polymorphism (rs number) (%)		Males (%)				Females (%)			
		Patients (n = 533)	Controls (n = 359)	?^2^	P	Patients (n = 461)	Controls (n = 349)	?^2^	P
apelin:	TT	−*	−			44.06	45.38		
rs3761581	TG	−	−			44.75	40.17	2.65	0.266
	GG	−	−			11.19	14.45		
	G	34.13	40.06	3.11	**0.077**	33.56	34.54	0.16	0.685
apelin:	AA	−	−			43.5	44.35		
rs56204867	AG	−	−			46.41	42.32	2.54	0.281
	GG	−	−			10.09	13.33		
	G	34.18	39.48	2.5	0.114	33.3	34.5	0.25	0.618
APJ:	GG	59.92	58.79			60.05	58.38		
rs7119375	GA	35.52	36.31	0.13	0.937	34.93	36.71	0.37	0.876
	AA	4.56	4.9			5.02	4.91		
	A	22.32	23.05	0.13	0.722	22.49	23.27	0.13	0.716
APJ:	CC	60.12	58.79			60.05	58.38		
rs10501367	CT	35.32	36.31	0.17	0.92	34.93	36.71	0.27	0.8758
	TT	4.56	4.9			5.02	4.91		
	T	22.22	23.05	0.16	0.686	22.49	23.27	0.13	0.716
APJ:	CC	58.53	57.64			59.13	55.78		
rs9943582	CT	34.92	35.73	0.07	0.966	33.01	35.55	0.91	0.635
	TT	6.55	6.63			7.76	8.67		
	T	24.01	24.5	0.05	0.817	24.32	26.45	0.93	0.335

* Because the gene encoding apelin is mapped on the X chromosome, genotype data are unavailable.

Signed and informed consent was obtained from each participant, and the project was approved by the ethics committee of Ruijin Hospital, Shanghai Jiao Tong University School of Medicine, and was conducted according to the Declaration of Helsinki Principles.

**Table 3:** The estimated frequencies of polymorphisms examined in apelin and APJ genes between CAD patients and controls according to the gender.

Haplotype	Males (%)			Females (%)		
	Patients	Controls	P†	Patients	Controls	P†
**apelin gene (rs56204867-rs3761581)**						
**A-T**	65.42	59.11	0.061	66.02	64.58	0.559
**G-G**	33.99	38.28	0.196	32.84	33.52	0.775
**G-T**	0.2	0.88	0.16	0.46	0.88	0.305
**A-G**	0.4	1.73	**0.047**	0.69	1.02	0.479
**APJ gene (rs7119375-rs10501367-rs9943582)**						
**G-C-C**	75.99	75.5	0.821	75.69	73.56	0.357
**A-T-T**	22.22	23.06	0.683	22.49	23.27	0.714
**G-C-T**	1.69	1.44	0.699	1.83	3.18	0.095

† P values were calculated after adjusting for age, type 2 diabetes mellitus, body mass index and systolic blood pressure.

### Index determination

At enrollment, onset age of CAD was recorded, and body weight and height were measured to calculate body mass index (BMI). Blood pressure was measured using a calibrated mercury sphygmomanometer with appropriate adult cuff size by certified examiners. Diagnosis of hypertension was based on the presence of elevated systolic (≥140 mmHg) and/or diastolic (≥90 mmHg) blood pressure, or current use of antihypertensive medications. All study participants were hypertensive patients. Type 2 diabetes mellitus was defined as a fasting plasma glucose level ≥7.0 mmol/L or non–fasting plasma glucose level ≥11.1 mmol/L, or taking hypoglycemic drugs or receiving parenteral insulin therapy.

**Table 4:** MDR analysis summary.

Best combination of each model	Cross-validation consistency	Testing accuracy	*P*
**Males**			
**rs3761581**	**8**	**0.5093**	**0.0408** *
rs3761581, rs9943582	5	0.4875	0.2418
rs3761581, rs56204867, rs9943582	9	0.5043	0.2956
rs3761581, rs56204867, rs10501367, rs9943582	7	0.5041	0.2304
rs3761581, rs56204867, rs7119375, rs10501367, rs9943582	10	0.4936	0.7106
**Females**			
rs3761581	10	0.5441	0.3001
**rs7119375, rs9943582**	**10**	**0.7318**	**<0.0001** *
rs56204867, rs7119375, rs9943582	9	0.7298	<0.0001
rs3761581, rs56204867, rs7119375, rs9943582	5	0.7029	0.0002
rs3761581, rs56204867, rs7119375, rs9943582, rs10501367	10	0.6654	0.0002

* The overall best MDR model.

Blood samples were collected after an overnight fasting from all participants. Plasma glucose was measured using an automated biochemical instrument (Beckman CX-7 Biochemical Autoanalyzer; Brea, CA). Plasma levels of triglyceride (TG), total cholesterol (TC), high-density lipoprotein cholesterol (HDL-C), low-density lipoprotein cholesterol (LDL-C), apolipoprotein A and B, blood urea nitrogen (BUN), creatinine (Cr), and urea acid (UA), were determined enzymatically using available kits and auto analyzer at the Department of Laboratory Medicine in Ruijin Hospital. Plasma high sensitivity C-reactive protein (hsCRP) levels were determined using a high-sensitivity ELISA kit (Biocheck Laboratories, Toledo, OH, USA) with a linear range of 0.62–119.3 mg/L and an inter-assay CV<7.5%.

### Genotyping

Genomic DNA was isolated from peripheral blood leukocytes by standard phenol-chloroform method, and was stored at −20°C until required for batch genotyping. Genotypes of five examined polymorphisms (Figure 1) were determined by PCR-LDR (ligase detection reactions) method as previously described [15]. The primers for amplification and the probes for LDR are available from the authors upon request. The PCR primers were synthesized by Shanghai Generay Biotech Co., Ltd., and PCR reactions were conducted in EDC-810 Amplifier (Dongsheng Innovation Biotech Co., Ltd., China). Cycling parameters were as follows: 94°C for 2 min; 35 cycles of 94°C for 20 s; 60°C for 20 s; 72°C for 20 s; and a final extension step at 72°C for 3 min.

For each polymorphism examined, two specific probes were synthesized to discriminate specific bases with one common probe labeled by 6-carboxy-fluorescein (FAM) at the 3′ end and by phosphorylated at the 5′ end. The multiplex ligation reaction was carried out in a reaction volume of 10 μl containing 2 μl of PCR product, 1 μl 10×Taq DNA ligase buffer, 1 μM of each discriminating probe, 5 U Taq DNA ligase, and the ligation parameters were 30 cycles of 94°C for 30 s and 56°C for 3 min. After reaction, 1 μl LDR reaction product was mixed with 1 μl ROX passive reference and 1 μl loading buffer, and then denatured at 95°C for 3 min, chilled rapidly in ice water. The fluorescent products of LDR were differentiated using ABI 3730XL sequencer (Applied Biosystems, USA).

### Statistical analysis

Data management and statistical analyses were conducted by STATA software 11.0 version for Windows (StataCorp LP, College Station, TX, USA). Because the gene encoding apelin is mapped on the X chromosome (only one copy), the results were stratified by gender. Skewed quantitative variables were transformed to achieve normality. Between-group variables were compared by unpaired Student's t-test or Mann-Whitney U test where appropriate, and genotype/allele frequencies were compared by χ^2^ test. The goodness-of-fit of the observed allele frequencies with the expected frequencies by Hardy-Weinberg equilibrium was assessed by χ^2^ test. Two-tailed P<0.05 was accepted as statistical significance.

The haplotype frequencies of examined polymorphisms in apelin and APJ genes were estimated by haplo.em program, which computes maximum likelihood estimates of haplotype probabilities using the progressive insertion algorithm which progressively inserts batches of loci into haplotypes of growing lengths. Simulated P values were calculated based on 1000 replicates. The program haplo.em was implemented in Haplo.Stats software (version 1.4.0) operated in the R language (version 2.14, available at the website http://www.r-project.org).

Interaction analysis was conducted in the open-source multifactor dimensionality reduction (MDR) software (version 2.0) (www.epistasis.org) [16], [17]. All possible combinations of examined polymorphisms were constructed using MDR constructive induction. Then a Bayes classifier in the context of 10-fold cross-validation was employed to estimate the testing accuracy of each best model. A single best model had maximal testing accuracy and cross-validation consistency as a measure of the number of times of 10 divisions of the data that the best model was extracted. Statistical significance was evaluated using a 1000-fold permutation test to compare observed testing accuracies with those expected under the null hypothesis of null association. Permutation testing corrects for multiple testing by repeating the entire analysis on 1000 datasets that are consistent with the null hypothesis. Further, to validate the effectiveness of MDR method, a classical logistic regression analysis was undertaken for the extracted best model in each gender. Risk prediction for CAD was estimated by odds ratio (OR) and 95% confidence interval (95% CI).

Study power was estimated by adopting PS Power and Sample Size Calculations software (version 3.0).

## Results

### Baseline characteristics

As shown in Table 1, there were similar distributions between CAD patients and controls in terms of BMI, SBP, and plasma levels of TC, LDL-C, BUN, and UA in overall population or within both genders (P>0.05). Compared with controls, CAD patients were slightly younger (P = 0.03) for males, and had higher levels of fasting glucose, LP(a) and hsCRP for both genders, higher levels of TG (P = 0.01) for females, but had lower levels of HDL-C and Apo A for both genders, and lower DBP (P = 0.007) for females.

### Single-locus analysis

Because apelin gene is mapped on X chromosome, Hardy-Weinberg equilibrium test of rs3761581 and rs56204867 was only performed in females. The genotype distributions of five examined polymorphisms followed Hardy-Weinberg equilibrium in control groups of both genders (P>0.05). The rs3761581-G allele frequency was slightly overrepresented in controls (40.06%) relative to patients (34.13%) in males (P = 0.077). Statistically, there was no significant difference in the genotype/allele distributions of examined polymorphisms between CAD patients and controls (Table 2).

### Haplotype analysis

Because apelin and APJ genes were assigned on different chromosomes, haplotype analyses were conducted separately. In males, low-penetrance haplotype A-G (in order of rs56204867 and rs3761581) in apelin gene was significantly overrepresented in controls (1.73%) relative to CAD patients (0.4%) (P = 0.047), whereas haplotype A-T was marginally higher in patients (65.42%) than in controls (59.11%) (P = 0.061) (Table 3). No significance was reached for apelin gene in females and for APJ gene in both genders, even after controlling age, type 2 diabetes mellitus, BMI and SBP. For haplotype A-G in apelin gene with marginal significance in prediction of CAD, the power to reject the null hypothesis of no difference in frequencies between patients and controls was 71.8%.

### Interaction analysis

An exhaustive MDR analysis that evaluated all possible combinations of five examined polymorphisms is summarized by gender in Table 4. Each best model was accompanied with its testing accuracy, cross-validation consistency and significant level as determined by permutation testing. In males, the overall best MDR model included rs3761581 in apelin gene, and this model had a maximal testing accuracy of 0.5093 and a cross-validation consistency of 8 out of 10 (P = 0.0408). In females, rs7119375 and rs9943582 in APJ gene constituted the overall best MDR model with a maximal testing accuracy of 0.7318 and a maximal cross-validation consistency of 10. This model was significant at the level of <0.0001, indicating that a model this good or better was observed only by less than one out of 1000 permutations and was thus unlikely under the null hypothesis of null association.

In addition, a logistic regression model was employed to validate the predictive value of the polymorphism(s) in the best MDR model in each gender. Interestingly and consistently, rs3761581 from the best overall model in males conferred a marginally significant role on the development of CAD (OR = 0.77; 95% CI: 0.57–1.02; P = 0.072) after adjusting for age, type 2 diabetes mellitus, BMI and SBP, and in females, interaction probability of rs7119375 and rs9943582 from the best overall model reached statistical significance (OR = 0.88; 95% CI: 0.81–0.95; P = 0.02), these findings further validating that from MDR method.

## Discussion

In this study, we sought to investigate the association of five promising polymorphisms in apelin/APJ pathway with CAD among 1702 Chinese hypertensive patients. Our principal findings demonstrated that although low-penetrance haplotype A-G in apelin gene exerted a contributive effect on the occurrence of CAD in males, interactive effects of genetic defects in apelin/APJ pathway might confer a potential risk for CAD in Chinese hypertensive patients. To the authors' knowledge, this is the pilot study exploring the genetic susceptibility of apelin/APJ pathway to CAD in Chinese.

Several strengths distinguishing the present investigation merit adequate consideration. First, this study was based on our previous findings linking the top polymorphic markers of apelin/APJ pathway to hypertension and its related phenotypes [10], [11], and inclusion of genetic markers were biologically plausible [18], [19], [20]. Although hypertension, as an independent predisposing factor, perpetuates CAD, our results supported a small to moderate contribution of apelin/APJ pathway to the development and progression of CAD among hypertensive patients. Second, all CAD patients enrolled were angiographically-confirmed with ≥70% stenosis in at least one of the three major coronary arteries or major branches. It is highly recommended that selection of extreme phenotypes enhances the power and chance of teasing out major contributing genes [21]. Third, our sampling of controls was also angiographically defined, the strategy that can minimize the misclassification of study participants as far as possible. Moreover, genotypes of examined polymorphisms respected the Hardy-Weinberg equilibrium, lowering the likelihood of being biased by faulty genotyping or population stratification. Fourth, data from 1702 study participants were analyzed with statistical adjustment for traditional confounders. Although residual confounding by incompletely measured or unmeasured physiologic covariates might exist in our results, it seems unlikely that our results could be explained by confounding.

Recently, in an Italian population, Falcone and coworkers genotyped two intronic polymorphisms (G212A and A445C) in APJ gene, and they failed to confirm their association with CAD [22]. Moreover, Hinohara and coworkers in the Japanese and Korean populations also failed to observe a positive association between rs9943582 in APJ gene and CAD [23], in agreement with the results of our single-locus analysis. Even though, we cannot rule out the participation of examined polymorphisms in the functional expression of apelin/APJ pathway or CAD, because the estimated low-penetrance haplotype in apelin gene exhibited significant association with CAD risk, which challenges the claim of the common-disease common-variant hypothesis [24]. Moreover, failure to validation is not uncommon in genetic studies of CAD, and it is assumed that this might be due to the failure to account for other important variables or the disregard of gene-gene and gene-environment interaction on a low-risk population. Therefore, large well-designed studies are warranted to confirm or refute our findings.

Albeit nonsignificant, our findings offered several hints for the gender-specific association of apelin/APJ pathway with CAD. For instance in males, haplotype A-G of apelin gene in males was overrepresented in controls relative to in CAD patients, but exhibited no differences in females. Similar tendency was noted for haplotype G-C-T of APJ gene in females, although no significance was attained. Further, our findings implicated the predominant role of apelin in males and that of APJ in females in predisposition to CAD, as indicated by the results of both novel MDR method and classical logistical regression analysis. Besides sexual chromosome differences between two genders, the gender-specific roles of lifestyle components or the X-chromosome-based gene-gene interaction (such as apelin and ACE2) may take in determining the disease risk [25]. Moreover, sex hormone might exert a direct action given the fact that compared with the age-matched males, premenopausal females are at lower risk for cardiovascular diseases, whereas postmenopausal women entail greater risk [26]. Nevertheless, besides the insufficient sample size, our findings not only implied a gender-specific association of apelin/APJ pathway with CAD in hypertensive patients, but also proved the effectiveness of MDR method effective in detecting and characterizing multilocus interactions among many different polymorphisms. Further genotyping data from apelin/APJ pathway, incorporating the haplotype and synergism analytical strategy, would facilitate the identification of individuals at high risk of developing CAD in future clinical screening.

Finally, interpretation of our results should be viewed in light of several limitations. First, this study was retrospective in design, which precludes further comments on causality. Second, we only involved genes in apelin/APJ pathway, and it is encouraged to investigate its relevance with other pathways, such as renin-angiotensin system [27]. Third, the sample size in this study was not large enough (994 CAD patients and 704 controls), especially upon stratification by gender, because as suggested to generate robust data, a much larger sample set involving >1000 participants in each group might be required [28]. Fourth, we recruited participants aged more than 50 years, and future larger association studies in a young population of CAD patients are of specific interest, because genetic factors may have greater contribution to those suffering premature CAD and in the absence of strong environmental risk factors [29]. Last but not least, the fact that our study subjects was of Chinese ancestry limited the generalizability of our findings, reinforcing future validation in other ethnics.

Despite these limitations, our findings demonstrate a contributive role of low-penetrance haplotype in apelin gene on CAD in males, and more importantly, interactive effects of genetic defects in apelin/APJ pathway might confer a potential risk in Chinese hypertensive patients. Nevertheless, for practical reasons, we hope that this study will not remain just another endpoint of research instead of a beginning to establish background data to further investigate the molecular mechanisms of apelin/APJ pathway and CAD.
